# Synchronous bilateral breast cancer: A first case with lobular and mucinous carcinoma

**DOI:** 10.1016/j.radcr.2023.05.004

**Published:** 2023-06-30

**Authors:** Soukaina El Amrani, Zahi Hiba, Abdelmoughit Hosni, Youssef Omor, Rachida Latib

**Affiliations:** Radiology Department, National Institute of Oncology, Ibn Sina Hospital, Mohammed V University, Rabat, Morocco

**Keywords:** Synchronous breast carcinoma, Lobular carcinoma, Mucinous carcinoma

## Abstract

This report describes a rare case of synchronous bilateral carcinoma of the breast in a postmenopausal female patient, with mucinous carcinoma measuring 5 cm in the left breast and multifocal multicentric lobular carcinoma in the right breast. The patient underwent a bilateral mastectomy with adjuvant chemotherapy.

To the best of our knowledge, this is the first report of a case of mucinous carcinoma and infiltrating lobular carcinoma coexisting in distinct breasts.

## Introduction

The incidence of bilateral breast cancer (BBC) has been reported to range from 0.3% to 12% [1].

A particularly unusual entity is the association of 2 distinct histology types.

Mucinous carcinoma is uncommon, usually affects postmenopausal women, and has a generally positive evolution. Lobular carcinoma is the second most common histological form after ductal carcinoma, accounting for 10%-15% of all breast cancer cases. We describe a case of a 69-year-old female patient who had a multifocal, multicentric lobular carcinoma and a mucinous carcinoma that occurred simultaneously and independently.

## Case report

A 56-year-old patient presented with bilateral palpable breast masses of 3-month duration.

On clinical examination, there were bilateral palpable breast masses that were hard, mobile, and irregular. On the left side, there was skin tethering.

Ultrasound and mammography of bilateral breasts demonstrated advanced bilateral breast cancer.

The right breast had a spiculated, high-density mass in the upper outer quadrant measuring 2.5 cm. An additional 7 smaller lesions were distributed through the right breast, consistent with satellite lesions. The largest pathological node in the right axilla measured 9 × 9 mm.

**Fig. 1:**
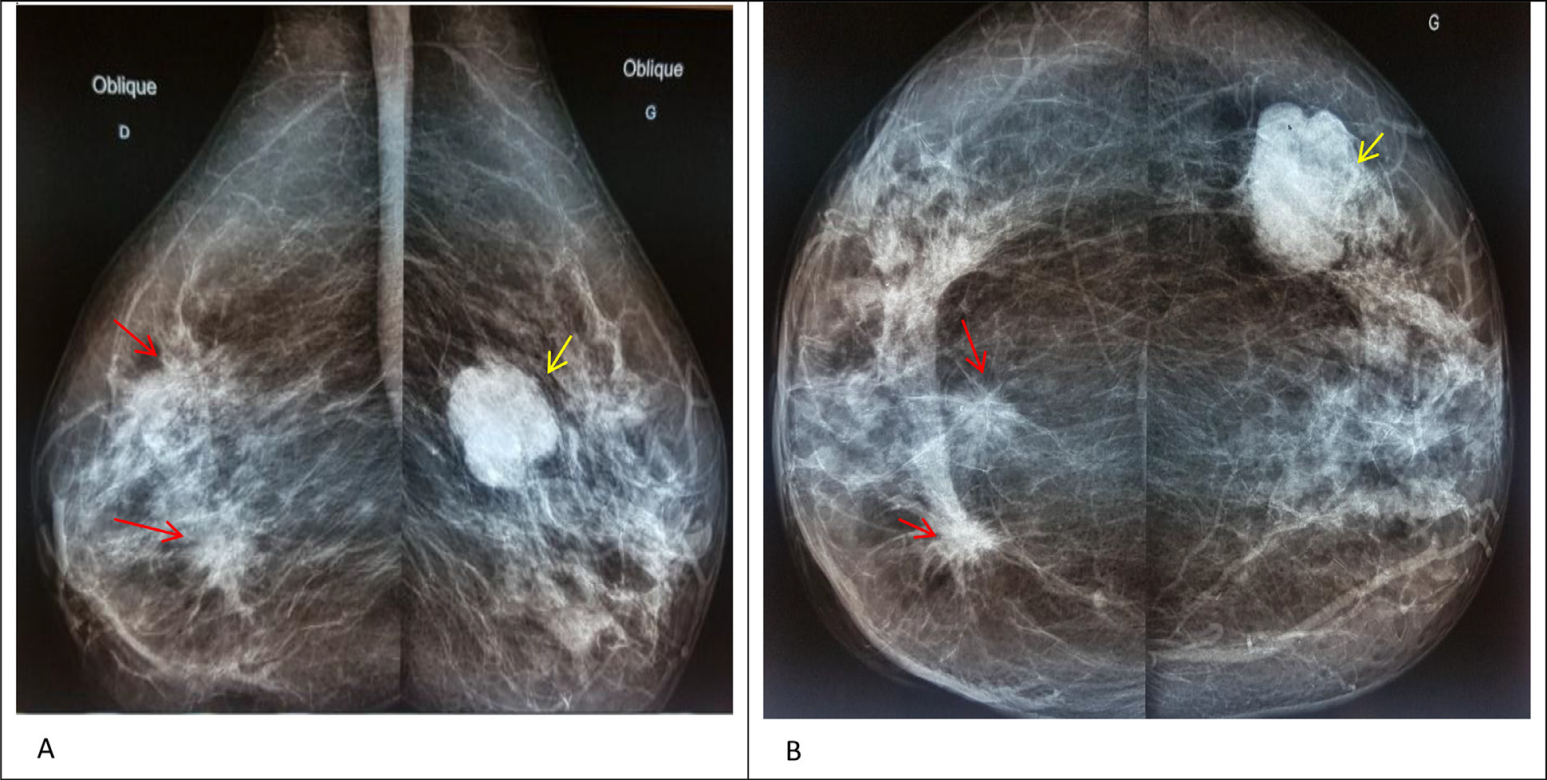
Mammogram craniocaudal (A) and mediolateral oblique (B) views of both breasts showing 2 high-density spiculated masses with focal calcifications in the upper outer quadrant and at the junction of the inner quadrants of the right breast (red arrow) and an ovoid, irregular, microlobulated mass, measuring 5.0 cm in diameter, in the upper outer quadrant of the left breast (yellow arrow).

**Fig. 2:**
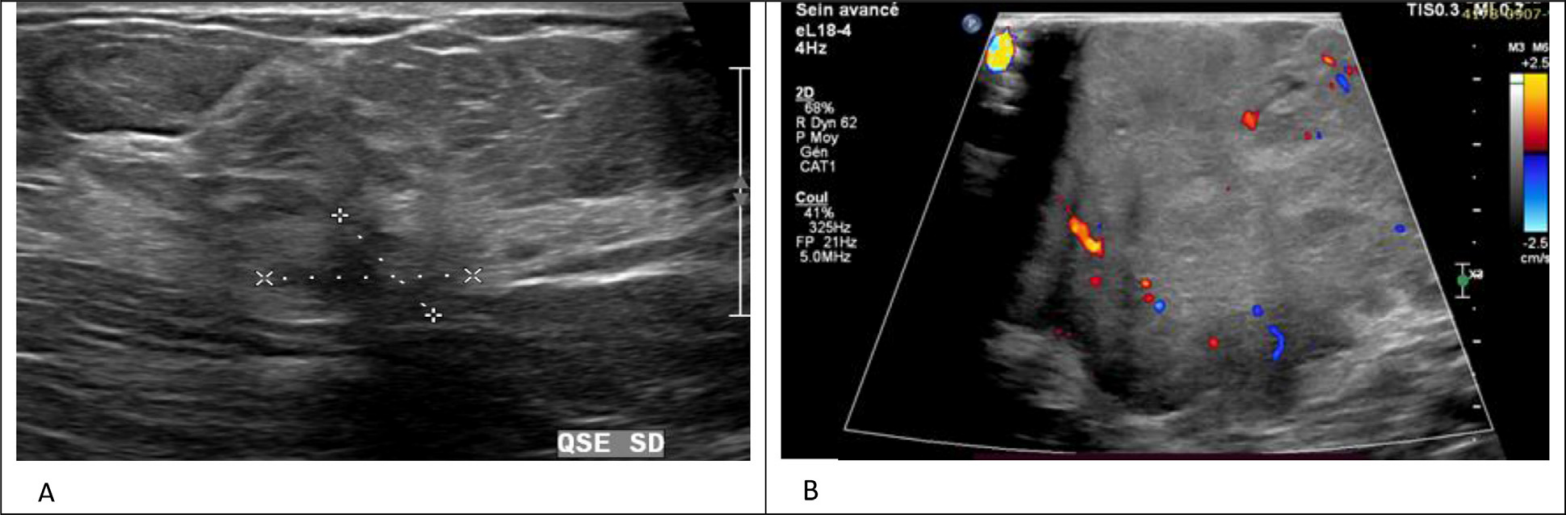
Ultrasonography of both breasts shows a spiculated, poorly circumscribed, hypoechoic mass with a posterior acoustic shadow in the left breast and an oval, irregular, heterogeneous mass with a diameter of 5 cm and rim vascularity in the left breast.

In the left breast, a 4 cm microlobulated mass is in the upper outer quadrant. The largest node in the left axilla measured 13 × 10 mm (Fig. 1). Complementary ultrasound revealed, in the left breast, a large heterogeneous hypoechoic mass with lobulated contour, acoustic shadowing, and infiltration of the skin. The ultrasound of her right breast found 2 hypoechoic masses, corresponding in size and location to the lesions seen on the right mammogram (Fig. 2), in addition to 2 abnormal-looking nodes in the left axilla; breasts classified as BIRADS 5.

A core biopsy from a lesion on the left breast demonstrated mucinous carcinoma, Scarff Bloom Richardson (SBR) II, expressing estrogen and progesterone hormone receptors but not expressing HER2.

Right core biopsies revealed an invasive lobular carcinoma, SBR grade II, expressing estrogen and progesterone hormone receptors but not expressing HER2.

**Fig. 3:**
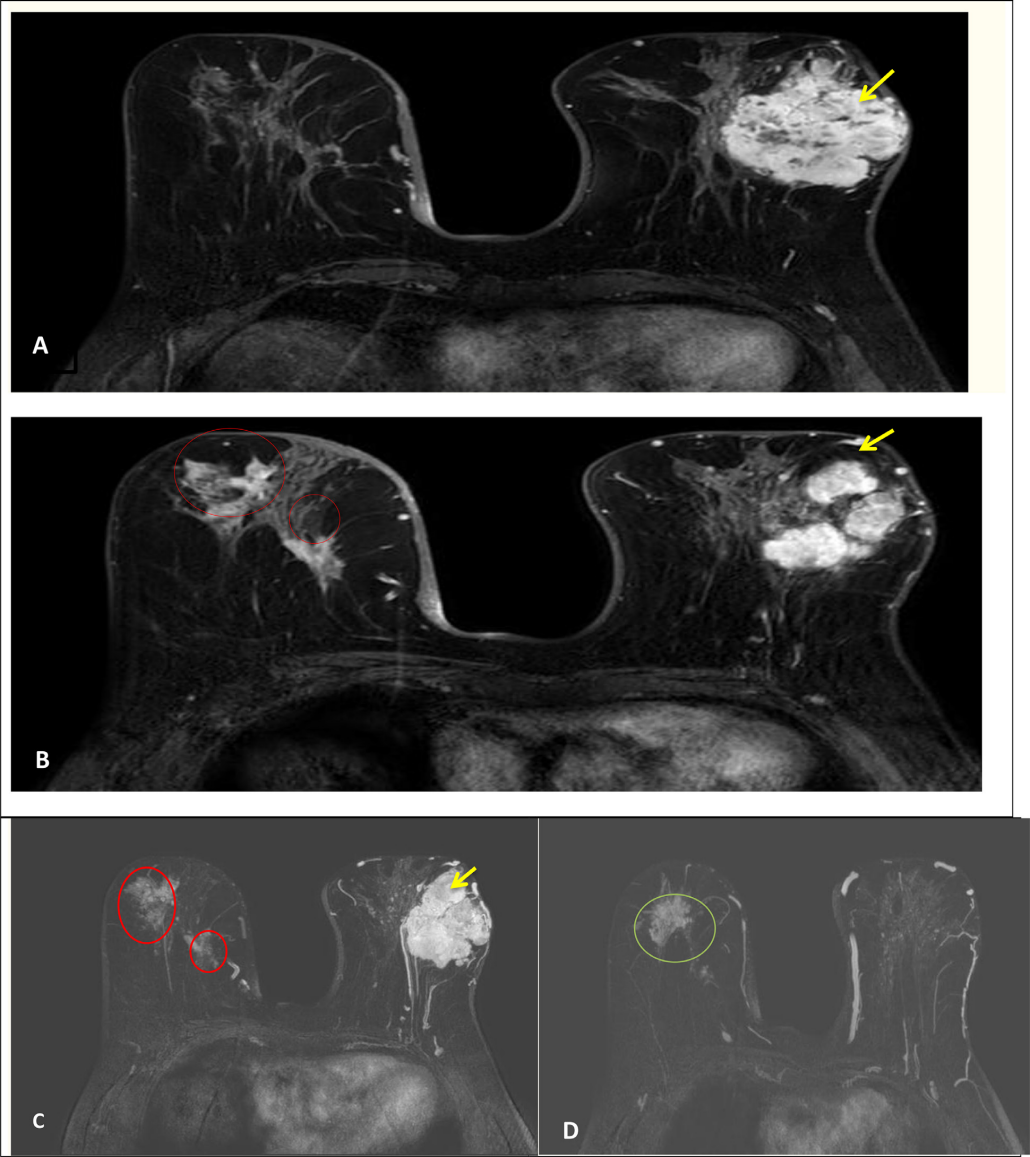
Axial postcontrast T1 FAT SAT (A, B) Axial 3D Maximal Intensity Projection (C, D) breast MRI showing in the left breast a lesion with irregular margins and heterogeneous enhancement (yellow arrow), in the right breast 2 lesions with spiculated margins (red circle), and MRI has shown multicentricity on the left upper outer quadrant (green circle).

The magnetic resonance imaging (MRI) showed a heterogeneous 7 cm x 6 cm mass with microlobulation occupying the entire left breast, enhancement with type 2 curve on postcontrast, with skin infiltration, and T2 hypointense true masses of the right breast, spiculated margins, and enhancement with type 2 curve (Fig. 3).

A work-up including thoracic-abdominal-pelvic CT-Scan (TAP CT-Scan) and bone scintigraphy was unremarkable.

Our patient underwent a bilateral mastectomy. The histopathology confirmed the 2 primary cancers.

The left breast was classified as T3N1M0 and the right as T2N0M0.

Postoperatively, the patient was on adjuvant chemotherapy and responded very well to the treatment.

## Discussion

According to the World Health Organization (WHO), breast cancer is the most common cancer in the world and the first cause of death from cancer.

The incidence of synchronous bilateral breast carcinoma (SBBC) depends on the criteria used to define it. For some, it is based on pathological findings, defining SBBC as a new primary cancer when it is histologically different from the first cancer [2].

Others define it as 2 independent primary lesions arising in both breasts within a maximum of a 6-month interval, in the absence of distant metastases [3], notion founded on a chronological basis.

Several publications propose that a family history of breast cancer, young age, a first lobular (in situ or invasive), ductal in situ, multifocal or multicentric type of breast cancer are risk factors for bilateral breast carcinoma (BBC) [4].

In accordance with the literature, hormone receptors were positive while HER was negative in our case. There is no clear relationship between estrogen receptor (ER)and progesterone receptor (PR) positivity and bilaterality of the tumor. But bilaterality is more commonly seen in cases with HER2/neu overexpression [5].

It is classically accepted by different authors that lobular carcinoma is associated with a high risk of multifocality (multiple lesions-same quadrant/>5 cm apart) or multicentricity (multiple lesions-usually different quadrants/<5 cm apart) and bilaterality [6,7]. In our case, 2 quadrants were affected with 2 lesions that were 2 cm apart.

Lesser et al. [8] found a high proportion of bilaterality in patients with multicentric cancer; Newman et al. [9] found that multifocality was predictive of bilaterality.

The histological study of tumors in the context of SBBC is an important element. The most common histological type found in the studies is invasive ductal carcinoma [10].

However, the incidence of invasive lobular carcinoma and the finding of lobular carcinoma in situ is slightly higher among synchronous bilateral carcinomas as compared to unilateral disease. Mucinous carcinoma is a particular type of tumor that represents between 1% and 7% of all invasive breast cancers.

There have been only a few case reports of SBBC of different histological types. To our knowledge, this is the first case of bilateral lobular and mucinous breast cancer.

An extremely rare form of breast cancer known as mucinous carcinoma (MC), also known as colloid or gelatinous carcinoma, is characterized by the formation of mucus and typically affects postmenopausal women [11].

Our patient belonged to the age group corresponding to the maximum incidence of MC of the breast.

The case presented here is unusual because bilaterality is exceptional in MC.

Histologically, 2 types are distinguished: pure and mixed MC, with different implications in diagnosis and prognosis [12].

Radiologically, invasive lobular carcinoma (ILC) usually appears as an irregular mass with hypoechoic, heterogeneous internal echoes, posterior acoustic shadowing, or as an architectural distortion.

The most common magnetic resonance imaging (MRI) presentation of ILC is that of a mass with irregular or spiculated margins, followed by a nonmass lesion in 20%-40% of cases [1,7].

Both mammography and ultrasound findings correlate with the histological type of the tumor and the extracellular mucin volume. For pure MC, it is a nodular mass that is circumscribed and multi-lobed with well-defined contours. The well-defined limits are correlated with the volume of extracellular mucin, so that they can be mistaken for benign formations. The mammographic aspect of mixed MC is nonetheless more suspected. It appears as a mass of irregular contours with poorly defined boundaries.

The MRI aspect of pure MC is characteristic. Indeed, the signal intensity in the T1 sequence after gadolinium injection varies according to the tumor concentration in extracellular mucin. In the T2 sequence, the lesion is characterized by intense and homogenous contrast enhancement and dynamic analysis by a fine increase of the signal and then plateau, unlike mixed MC, which presents a heterogeneous enhancement with a washout appearance [13].

The management of patients with SBBC consists of treating the 2 breasts as separate diseases. In addition, the occurrence of contralateral breast cancer is not a contraindication to breast conservation.

In our patient, a bilateral mastectomy with axillary node clearance was used as a surgical therapeutic approach followed by adjuvant chemotherapy.

Numerous studies have demonstrated that the prognosis of BBC is comparable to that of unilateral breast cancer, and that the prognosis is mostly correlated with the stage of the most advanced tumor.

In conclusion, to the best of our knowledge, this is the first report to describe a case of synchronous coexistence of mucinous and multifocal, multicentric lobular carcinoma in a distinct breast.

## Patient consent

Written informed consent for publication was obtained from patient.

## References

[1] Krishnappa R, Chikaraddi SB, Deshmane V (2014). Primary synchronous bilateral breast cancer. Indian J. Cancer.

[2] de la Rochefordiere A., Asselain B., Scholl S. (1994). Simultaneous bilateral breast carcinomas: a retrospective review of 149 cases. Int. J. Radiat. Oncol. Biol. Phys..

[3] Chaudary M.A., Millis R.R., Hoskins E.O.L. (1984). Bilateral primary breast cancer: a prospective study of disease incidence. J. Br. Surg..

[4] Marpeau O., Ancel P.-Y., Antoine M. (2008). Cancers du sein bilatéraux synchrones: facteurs de risque, diagnostic, histologie, traitement. Gynecol. Obstet. Fertil..

[5] Kappikeri V.K.S., Kriplani A.M. (2015). Bilateral synchronous carcinoma breast-a rare case presentation. Springerplus.

[6] Chaudry M.A., Winslet M.C. (2009). Surgical oncology.

[7] Farrokh A., Goldmann G., Meyer-Johann U. (2022). Clinical differences between invasive lobular breast cancer and invasive carcinoma of no special type in the german mammography-screening-program. Women & Health.

[8] Lesser M.L., Rosen P.P., Kinne D.W. (1982). Multicentricity and bilaterality in invasive breast carcinoma. Surgery.

[9] Newman L.A., Sahin A.A., Bondy M.L. (2001). A case–control study of unilateral and bilateral breast carcinoma patients. Cancer: Interdisciplinary International Journal of the American Cancer Society.

[10] Tousimis E. (2005). Synchronous bilateral invasive breast cancer. Breast Cancer.

[11] Marrazzo E., Frusone F., Milana F. (2020). Mucinous breast cancer: a narrative review of the literature and a retrospective tertiary single-centre analysis. Breast.

[12] Chaudhry A.R., El Khoury M., Gotra A. (2019). Imaging features of pure and mixed forms of mucinous breast carcinoma with histopathological correlation. Brit. J. Radiol..

[13] Korbi A., Mhabrech H., Farouk E., Cherif O., Daldoul A., Hafsa C. (2018). Mucinous Breast Carcinoma: Anatomo-Clinical Radiological and Therapeutic Features. Open Access Libr..

